# Effects of Simulated Human Gastrointestinal Digestion of Two Purple-Fleshed Potato Cultivars on Anthocyanin Composition and Cytotoxicity in Colonic Cancer and Non-Tumorigenic Cells

**DOI:** 10.3390/nu9090953

**Published:** 2017-08-29

**Authors:** Stan Kubow, Michèle M. Iskandar, Emiliano Melgar-Bermudez, Lekha Sleno, Kebba Sabally, Behnam Azadi, Emily How, Satya Prakash, Gabriela Burgos, Thomas zum Felde

**Affiliations:** 1 School of Human Nutrition, McGill University, 21,111 Lakeshore, Ste. Anne de Bellevue, QC H9X 3V9, Canada; michele.iskandar@mail.mcgill.ca (M.M.I.); emiliano.melgar-bermudez@mail.mcgill.ca (E.M.-B.); kebba.sabally@mcgill.ca (K.S.); behnam.azadi@mcgill.ca (B.A.); emily.how@mail.mcgill.ca (E.H.); 2 Chemistry Department, Université du Québec à Montréal, P.O. Box 8888, Downtown Station, Montreal, QC H3C 3P8, Canada; sleno.lekha@uqam.ca; 3 BioMedical Engineering Department, McGill University, 3775 University Street, Room 311, Montreal, QC H3A 2B4, Canada; satya.prakash@mcgill.ca; 4 International Potato Center (CIP), Avenida La Molina 1895, Apartado Postal 1558, Lima, Lima 12, Peru; g.burgos@cgiar.org

**Keywords:** purple-fleshed potato, anthocyanins, biotransformation, human gastrointestinal model, antioxidant, cancer cells, cytotoxicity

## Abstract

A dynamic human gastrointestinal (GI) model was used to digest cooked tubers from purple-fleshed Amachi and Leona potato cultivars to study anthocyanin biotransformation in the stomach, small intestine and colonic vessels. Colonic Caco-2 cancer cells and non-tumorigenic colonic CCD-112CoN cells were tested for cytotoxicity and cell viability after 24 h exposure to colonic fecal water (FW) digests (0%, 10%, 25%, 75% and 100% FW in culture media). After 24 h digestion, liquid chromatography-mass spectrometry identified 36 and 15 anthocyanin species throughout the GI vessels for Amachi and Leona, respectively. The total anthocyanin concentration was over thirty-fold higher in Amachi compared to Leona digests but seven-fold higher anthocyanin concentrations were noted for Leona versus Amachi in descending colon digests. Leona FW showed greater potency to induce cytotoxicity and decrease viability of Caco-2 cells than observed with FW from Amachi. Amachi FW at 100% caused cytotoxicity in non-tumorigenic cells while FW from Leona showed no effect. The present findings indicate major variations in the pattern of anthocyanin breakdown and release during digestion of purple-fleshed cultivars. The differing microbial anthocyanin metabolite profiles in colonic vessels between cultivars could play a significant role in the impact of FW toxicity on tumor and non-tumorigenic cells.

## 1. Introduction

Potatoes are a versatile sustainable food staple in many countries providing populations with an affordable source of key phytonutrients and valuable income for growers. Colored fleshed potatoes contain anthocyanins, which are red, blue and purple water soluble pigments that occur in the glycosylated form with one or more sugar moieties [1]. Anthocyanins are glycosides or acylglycosides of six common dietary aglycone anthocyanidins, which consist of pelargonidin, cyanidin, peonidin, delphinidin, petunidin and malvidin. Anthocyanin structures vary by glycosidic substitution and acylation of the sugar groups with acids that can include acetic acid, *p*-coumaric acid, caffeic acid, malonic acid, sinapic acid, ferulic acid, oxalic acid and succinic acid [2]. Glucose, xylose, galactose, arabinose and rhamnose are common monosaccharides in anthocyanins attached to the C3 position of the flavan structure, whereas common diglycosides found in anthocyanins include rutinose (glucose and rhamnose) and sophorose (glucose and glucose).

There is great interest in the food content of anthocyanins due to their proposed chemopreventative properties towards several disease processes such as atherosclerosis and retinal light-induced damage [1]. Anthocyanins are present in the flesh and skin of several purple and red fleshed potatoes, which show a wide range of anthocyanin structures and concentrations that are largely cultivar-dependent [3] and location-dependent [4]. The anthocyanin content of colored fleshed potatoes has been related to a two- to three-fold greater antioxidant potential than white-fleshed potatoes [1,3]. Boiled purple-fleshed potatoes are a good source of anthocyanins and show high antioxidant capacity [1], although the effect of boiling on anthocyanin content can also be variety-dependent [5]. Since potato is an important food staple that is considered the most important vegetable crop worldwide, the contribution of potatoes towards anthocyanin intake can be significant.

There is fundamental information lacking regarding how the anthocyanin structures and bioactivities are affected during digestion of pigmented potatoes. Such knowledge can ultimately lead to enhancement of the chemopreventative properties of colored fleshed potatoes for consumers via breeding practices favoring genetic variants with desirable anthocyanin profiles. Anthocyanins are released from the food matrix and undergo biotransformation during the gastrointestinal digestive processes involving pH changes and digestive enzymes. In addition, they undergo major degradation via their metabolism by colonic microflora [6]. Simulated gastrointestinal digestion studies that include microbial batch cultures demonstrate major decreases in anthocyanins after exposure to pancreatic enzymes with an additional decline in anthocyanins after human fecal microbial metabolism [7,8]. Computer-controlled dynamic multistage continuous digestion models involving the ascending colon, transverse colon and descending colon vessels more closely simulate in vivo conditions due to pH adjustment of each colonic bioreactor, allowing for the development of varying microbial communities with different metabolic activities in each colonic vessel [9,10,11]. Varying results have been observed with respect to antioxidant capacity measures following simulated gut digestion of anthocyanin-rich foods with reports showing either a diminishment [12] or enhancement [13] of antioxidant activity post-digestion. Simulated digestion studies of anthocyanin-rich purple-fleshed potatoes that include the microbial and digestive conditions associated with different colonic segments of the gastrointestinal tract have not been performed to examine changes in anthocyanin structure or antioxidant capacity.

Dietary anthocyanins are implicated in the protective effects of fruits and vegetables against cancer. Anthocyanin-rich fractions and extracts from blueberry [14], bilberries [15,16], blackberries [17], chokecherry [18], java plum [19], sweet potato [20] and tart cherries [21] have been shown to effectively inhibit the growth of a variety of human colonic cancer cell lines. Extracts of anthocyanin-containing tart cherries [21,22], black raspberries [23] and purple corn [24] have been demonstrated to also inhibit tumor development in different animal colon cancer models. In terms of anthocyanin-rich potatoes, extracts from Bora Valley, Purple Majesty, Mountain Rose, Northstar and the wild species *Solanum pinnatisectum* exhibited inhibition of the growth of cultured human malignant cells [25]. The anti-proliferative properties of purple-fleshed potato extracts were also seen in HCT-116 and HT-29 colon cancer lines, regardless of prior baking or chip processing [26]. Despite such encouraging findings there has been limited research regarding how anti-carcinogenic activity is affected by changes in anthocyanin structures caused by digestive processes. A recent study showed that pepsin-pancreatin digests of the high anthocyanin-containing cv. Vitelotte noire purple potatoes were associated with diminished cell viability in the Caco-2 colon cancer cell model [27]. To our knowledge, the anti-cancer impact of colonic microbial anthocyanin metabolites generated from the digestion of anthocyanin-rich foods has not been previously addressed.

The first objective of this study was to evaluate the biotransformation of anthocyanins in cooked samples of the two purple-fleshed potato cultivars Amachi and Leona after digestion in the Computer Controlled Dynamic Human Gastrointestinal Model (GI model). Samples of both cultivars underwent digestion via the GI model and liquid chromatography-electrospray ionization-time-of-flight (LC-ESI-TOF) mass spectrometry (MS) was used to assess anthocyanin profiles and antioxidant capacity measures following digestive processes in compartments of the GI model (stomach, small intestine, ascending, transverse and descending colon). The second objective was to compare the effects of FW digests of the two cultivars obtained from the colonic reactors from the GI model on the cytotoxicity and cell viability on the human colonic adenocarcinoma Caco-2 cell line and normal colonic epithelial cells (CCD-112CoN).

## 2. Materials and Methods 

### 2.1. Plant Material

**Figure 1:**
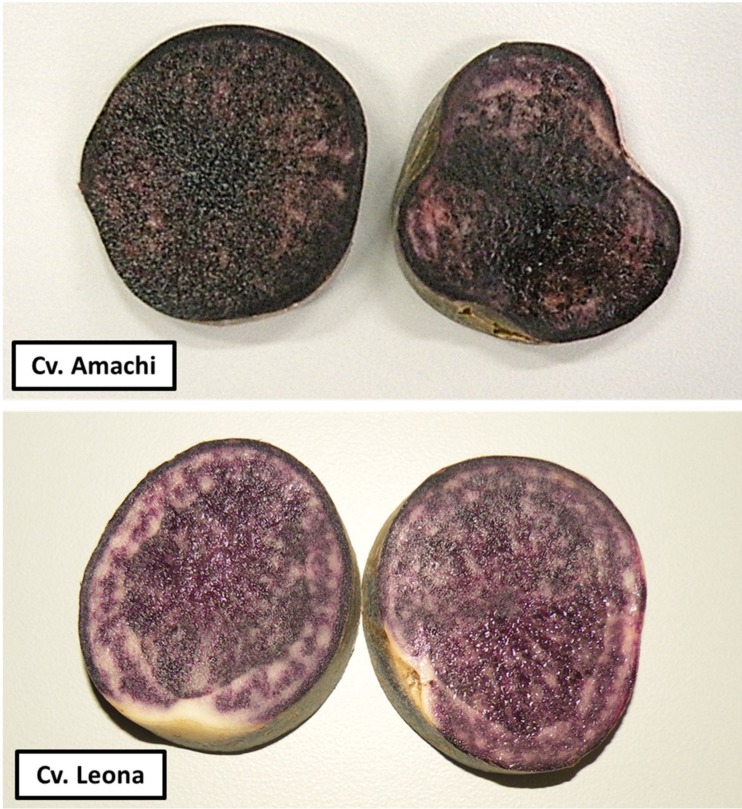
The purple-fleshed potato cultivars Amachi and Leona.

Tubers from two intensive purple-fleshed cultivars, Amachi and Leona (Figure 1), grown in Andahuaylas, Apurimac, Peru, were used in this study. Andahuaylas is located at 2926 meters above sea level in the Peruvian Andes. Both cultivars were selected based on their high total anthocyanin content (360 mg/100 g and 180 mg/100 g, respectively) and high antioxidant activity (945 mg Trolox equivalent/100 g and 542 mg Trolox equivalent/100 g, respectively) expressed on a fresh weight basis [28]. One hundred tubers from each cultivar were processed by the Quality and Nutrition Laboratory at the International Potato Center (CIP) in Lima, Peru, where representative tubers were cooked, peeled, freeze dried and milled through 40 mesh following the procedure described by Porras et al., 2014 [29]. Freeze dried and milled samples of each cultivar were sent to the School of Human Nutrition, McGill University, Canada.

### 2.2. Computer Controlled Dynamic Human Gastrointestinal Model

The simulated human GI model consisted of five consecutive reactors that represent the stomach (V1), small intestine (V2), the ascending (V3), the transverse (V4) and the descending colon (V5) that are interconnected by plastic tubing and peristaltic pumps as previously described [11]. The system is fully computer-controlled (LabVIEW^®^ software, National Instruments, Austin, TX, USA) for the addition of food to V1 and buffers to adjust pH of all compartments and pancreatic juice to V2. The pH was measured with a probe connected to a pH meter and was automatically adjusted to keep a pH of 2.0 in V1 and 6.5 in V2 via addition of 0.2 M NaOH or 0.5 M HCl. The flow of intestinal content between reactors was automatically computer controlled with a transit time of 2 h in each of the V1 and V2 compartments followed by 4 h digestion in the colonic vessels. The volume in the V1 and V2 reactors was 200 mL and the V3, V4 and V5 reactors had volumes of 500, 800 and 600 mL, respectively. Temperature-controlled water flowed between the double glass jacketed reactors to keep the temperature at 37 °C. The passage of food in the stomach was simulated by the addition of gastric solution including 0.1 M HCl and pepsin (P7125, Sigma-Aldrich, Oakville, ON, Canada). Upon entering V2, pancreatic juice supplemented with bile (12 g/L NaHCO, 0.9 g/L pancreatin; P 1750, Sigma-Aldrich) and 6 g/L Oxgall (DF 0128-17-8, Fisher Scientific, Ottawa, ON, Canada) were added to neutralize stomach acidity. The colonic vessels V3, V4 and V5 were pH-controlled between 5.6 and 5.9; 6.1 and 6.4; and 6.6 and 6.9, respectively. Freshly collected fecal samples from five non-smoking, healthy volunteers (3 males, 2 females aged 30–65 with BMI between 20 and 24.99 kg/m^2^) with no history of GI disease or antibiotic use in the previous 6 months were pooled and used to prepare a 10% fresh fecal slurry in sterile phosphate-buffered saline solution that inoculated the three colonic reactors. The fermentation vessels were kept at 37 °C and were stirred continuously on magnetic stirrers. The colonic reactors were maintained anaerobic by daily purging for 20 min into the headspace with oxygen-free nitrogen gas. For the bacterial populations from the fecal slurry to stabilize, the system was allowed to run for two weeks before the addition of the potato meal (stabilization period). During this time and during potato meal digestion, the system was supplied with 300 mL of GI nutrient solution at 8-h intervals, or three times daily. Prior to use, the solution was adjusted to pH 2, autoclaved, and stored at 37 °C. The composition of the GI nutrient solution was composed of arabinogalactan (1 g/L), pectin (2 g/L), xylan (1 g/L), glucose (0.4 g/L), yeast extracts (3 g/L), peptone (1 g/L), mucin (4 g/L), all of which were purchased from Sigma-Aldrich; starch (3 g/L) and cysteine powders (0.5 g/L), both of which were purchased from Fisher Scientific; this nutrient solution was previously shown to stabilize the microbial community in the colonic vessels [11]. This approach has been validated using enumeration procedures, short chain fatty acid production patterns, enzymatic activities, gas production, and by microorganism-associated activities [9,10,11].

After the 2-week stabilization period, the potato meal digestion was started in the GI model that consisted of 18.5 g of the cooked, freeze-dried, and milled phenolic-rich cultivars Amachi and Leona. On the day of treatment, the freeze-dried potato tuber samples were incorporated into the GI food solution and subjected to three 8 h cycles of digestion by the GI model. The calculated amount of potato meal provided to the gut model was based on the dry matter content of cooked freeze-dried potato (25.36%) according to the United States Department of Agriculture food database. The meal provided approximately one-half of the single serving of potato, which reflects typical daily intake for one cultivar as part of a daily mixed potato cultivar intake in Peru (80 kg/year) [30].

Aliquots (20 mL) were collected from all the vessels of the GI model before addition of the freeze-dried potato meal (*t* = 0 control) and after 8 h (*t* = 8) and 24 h (*t* = 24) of digestion. One day of treatment was followed by a 3-day washout period when the system was fed the control GI nutrient solution without potato. FW was prepared from the aliquots via centrifugation at 200× *g* for 20 min and the supernatants were stored at −80 °C for later analysis. To prevent photodecomposition, all digestive compartments and collection vessels were wrapped in tin foil.

### 2.3. LC-ESI-TOF-MS Analysis

Samples were thawed, spiked with 10 μg/mL keracyanin chloride internal standard, vortexed, filtered with 25 mm syringe filters (0.2 μm, MCE, sterile; 09-719C, Fisher Scientific) into high-performance liquid chromatography (HPLC) vials for ESI-TOF-MS analysis, which was conducted based on a modified method of Tian et al., 2005 [31]. Sample analysis was carried out using an Agilent 1200 series HPLC system equipped with an Agilent 6210 (LC-ESI-TOF)-(MS) (Agilent, Santa Clara, CA, USA). Anthocyanins were separated using gradient conditions on a Gemini-NX column (5 μm, 100 mm × 4.6 mm) (Phenomenex, Torrance, CA, USA). Elution involved mobile phases A (water + 0.1% formic acid (FA)) and B (acetonitrile + 0.1% FA). Initially, the mobile phase composition was held at 5% B for 1 min, then increased to 25% B at 30 min, with a sharp increase to 80% at 31 min held for 3 additional minutes prior to re-equilibration time back at the initial conditions. Solvent flow rate of 1 mL/min was used and 20 μL of sample was injected. Accurate mass data were obtained by both positive and negative ion ESI, which involved injection via two different methods. Data was acquired over a mass (*m*) to charge (*z*) ratio (*m*/*z*) range of 100 to 1000. The settings of tuning parameters were: gas flow 12 L/min, temperature 350 °C, capillary voltage (+/−) 4000 V, skimmer voltage 60 V, fragmentor 100 V and nebulizer 50 psi (344.74 kPa). The reference masses for internal calibration of the high resolution mass spectra were *m*/*z:* 121.050873, 922.009798 for the positive mode and *m*/*z:* 119.03632, 966.000725 for the negative mode. The acquisition of mass spectra was carried out using the MassHunter acquisition software (Agilent Technologies, software version 4.01b) and further processing was done using MassHunter Qualitative Analysis. Anthocyanins in the samples were identified based on their masses and information obtained from the literature. Anthocyanins were quantified using an internal standard keracyanin chloride (cyanidin-3-O-rutinoside chloride) and anthocyanin concentrations were expressed as keracyanin chloride equivalents (KCC-Eq). Extracted ion chromatograms of accurate masses for protonated (MH^+^) ions were used for confirmation of presence of parent anthocyanin compounds as well as metabolites within ± 10 ppm.

### 2.4. Ferric Reducing Antioxidant Power Assay

The ferric reducing antioxidant power (FRAP) assay described by Benzie and Strain (1996) [32] was used to determine the total antioxidant potential of the supernatant of collected FW samples. The FRAP reagent was prepared in a 10:1:1 ratio of 300 mM acetate buffer (pH 3.6) of 10 mM 2,4,6-tripyridyl-s-triazine solution, 40 mM HCl at 50 °C, and 20 mM FeCl_3_·6H_2_O solution. Once the FRAP working solution was prepared it was immediately incubated for 10 min at 37 °C. The reaction was carried out using a 96-well plate filled with 10 μL of sample or standard, 30 μL of H_2_O and 200 μL of the FRAP working solution for 30 min. The absorbance was read at 593 nm in a microplate reader (Infinite PRO 200 series, Tecan Group, San Jose, CA, USA). Ferrous sulfate solution was used as an external standard with a calibration curve range of 0.1 to 10 mM. The results were expressed as ferrous sulfate equivalents.

### 2.5. Cytotoxic Effects of FW with and without Purple Potato Digests on Caco-2 and CCD112-CoN Cells

The colonic adenocarcinoma Caco-2 cells and CCD-112CoN cells were obtained from American Type Culture Collection (Manassas, VA, USA). Cell culture medium was obtained from Invitrogen (Carlsbad, CA, USA). Caco-2 cells were cultured in Dulbecco’s modified Eagle’s Minimum Essential Medium (EMEM, pH 7.4) supplemented with 20% heat inactivated fetal bovine serum (FBS) and 1% (*v*/*v*) penicillin–streptomycin. CCD-112CoN cells were cultured in EMEM and 10% FBS. Both cell lines were grown in a humidified atmosphere containing 5% CO_2_ and 95% air at 37 °C. For cytotoxicity experiments, Caco-2 cells and CCD-112CoN cells were seeded on 24-well plates at cell densities of 5.0 × 10^5^ and 7.6 × 10^4^ cells per well, respectively [33] and incubated under the same atmospheric conditions for 24 h or until confluent. The cells were then treated with either FW alone or FW containing in vitro digests of the cvs. Amachi and Leona. The digests were pooled from the ascending, transverse and descending colon vessels from the human simulated gut model in the studies described above. Digests were filter-sterilized and the pH was adjusted to 7 using 0.1 N HCl or 0.1 N NaOH. The digests were mixed with EMEM (2% FBS) at six treatment concentrations (0%, 10%, 25%, 75% and 100% FW in a total volume of 500 μL per well) that were administered to the cells for 24 h. The 0% dose consisted of EMEM (2% FBS) only. Lactate dehydrogenase (LDH) released into the culture medium was used as an indicator of cell viability. Cell-free supernatants were collected and LDH was assessed using the Cytotoxicity Detection Kit (Roche Diagnostics, Laval, QC, Canada) according to the manufacturer’s instructions. LDH production from damaged cells was expressed as percent of LDH produced by untreated cells. LDH release is a reliable method for assessment of the extent of cell death regardless of type of cell death [34]. To compare the effects of the two cultivars on tumor cell viability, the 3-(4,5-dimethylthiazol-2-yl)-2,5-diphenyl tetrazolium bromide (MTT) assay [35] was performed with Caco-2 cells. Briefly, the cells were washed with PBS following the 24 h incubation with the digests and incubated for 3 h with MTT solution (0.5 mg/mL). The purple formazan crystals produced by viable metabolically active cells were then dissolved using a lysis solution (0.4 M HCl in 100% isopropanol) and absorbance was measured at 540 nm. Cell viability was expressed as percent of untreated control. Cell viability values regarding FW from the two cultivars were also compared based on IC_50_ values. Each treatment was administered in duplicate and four independent experiments were conducted. Statistical analyses were performed using SigmaPlot v. 13 (Systat Software Inc., Chicago, IL, USA). The cytotoxic effects of cvs. Amachi and Leona in vitro digests on the two cell lines were analyzed by two-way analysis of variance (ANOVA) using dose and cell line as main factors followed by Tukey’s post-hoc test for multiple comparisons. For the MTT assay, data were analyzed by two-way ANOVA using cultivar and dose as main factors followed by Tukey’s post-hoc test for multiple comparisons. The IC_50_ values were compared using Student’s *t*-test. Data are represented as mean of four independent experiments ± SE. Differences were considered to be significant at *p* < 0.05.

## 3. Results

### 3.1. Anthocyanins in Digested Amachi and Leona Cultivars

The majority of anthocyanins in potatoes have been shown to possess the structure: anthocyanidin 3-acyl-rutinoside-5-glucoside. The most common acyl substitutes are caffeoyl, *p-*coumaroyl or feruloyl residues [36,37]. This is in agreement with the data in Table 1 where these anthocyanin structures were predominant in V1 for both tested potato cultivars.

The total anthocyanin content measured throughout the GI model vessels differed greatly between the two cultivars as Amachi contained greater than 30-fold higher content. The overall percent composition of anthocyanin concentrations of Amachi in the GI model consisted of 70% petunidins, 20.8% peonidins, 8.9% cyanidins and 0.3% pelargonidins. In contrast to the predominant presence of petunidin in Amachi, the most abundant anthocyanins in Leona were cyanidin based compounds constituting 48.9% of the species found throughout the gastrointestinal model, followed by a 17.8% of peonidin based compounds, 17.7% pelargonidins and 15.6% petunidins.

The anthocyanin compound clearly identified to be present at the highest concentration in V1 for Amachi was petanin (petunidin 3-*p*-coumaroyl-rutinoside-5-glucoside (*m*/*z* 933)), which was found at concentrations several-fold higher than any other anthocyanin accounting for 67.5% of the total anthocyanin content. The relatively high abundance of petanin in the Amachi cv. has been previously reported in other pigmented potato cultivars including Hermanns Blaue, Highland Burgundy Red, Shetland Black and Vitelotte [38] as well as four purple or dark fleshed tetraploid Andigenum potato cultivars [36]. The presence of the aglycones cyanidin (*m*/*z* 287.01), peonidin (*m*/*z* 301.03) and petunidin (*m*/*z* 301.07) [36] in V1 of Amachi corresponds to their presence noted previously in purple potatoes [36,39]. Cyanidin was also noted in V1 for the Leona sample and accounted for 21% of the total anthocyanin content in the GI model. Peonanin (peonidin-3-*p*-coumaroyl-rutinoside-5-glucoside (*m*/*z* 917.27) and petunidin-3-rutinoside-5-glucoside (*m*/*z* 787.22) that were noted in V1 of the Amachi and Leona cultivars have been identified in purple-fleshed cultivars [36,39,40].

For Amachi, the concentration of most anthocyanin species was found to be highest in V1, which contained 97% of the total anthocyanins found throughout the GI model. The aglycones cyanidin and petunidin were only found in V1, while peonidin was observed in both V1 and V3. The Leona cv. contrasted greatly with the Amachi cv. in terms of the distribution of anthocyanin species in the GI model as only 46% of the total anthocyanins were present in the V1 vessel. The remaining anthocyanins were present primarily in the V4 and V5 colonic vessels that comprised 35% of the total anthocyanins. For both cultivars, a biphasic pattern in anthocyanin concentrations was observed in terms of lowered anthocyanin concentrations in V2 followed by increased amounts in the subsequent colonic vessels, which has been previously seen during anthocyanin digestion [41,42]. Anthocyanins are likely to be unstable in the V2 intestinal vessel due to their chemical decomposition at neutral pH [43], which is known to occur before subsequent exposure to colonic microbial metabolism [44]. Degradation of anthocyanins that has ranged from 30 to 80% has been reported in pancreatic in vitro digestion studies [7,45]. Significant anthocyanin losses after intestinal digestion have been observed previously with simulated GI digestion model studies of plant foods such as mulberry molasses and pestil [46] and red cabbage [8].

While most anthocyanins glycosylated at 3’O and 5’O in Amachi were present in V1, pelargonidin 3-glucoside (*m*/*z* 433.11), cyanidin 3-sophoroside-5-glucoside (*m*/*z* 773.21) and cyanidin 3-*p*-hydroxybenzoyl-sophoroside-5-glucoside (*m*/*z* 893.23) were only present in other vessels, which could indicate that their release from the food matrix occurs later in digestion. There were six other species that were not seen in the V1 vessel for Amachi, which included cyanidin 3-(6-caffeoyl-glucoside) (*m*/*z* 611.14), cyanidin 3-sophoroside (*m*/*z* 611.16), peonidin 3-(6-*p*-coumaroyl-glucoside) (*m*/*z* 612.14), cyanidin 3-(6”-caffeoyl-sophoroside) (*m*/*z* 773.19), cyanidin 3-(6”-caffeoyl-6”-*p*-hydroxybenzoyl-sophoroside) (*m*/*z* 893.21) and peonidin 3-caffeoyl-*p*-hydroxybenzoyl-sophoroside (*m*/*z* 907.22). These species predominantly first appeared in the V3 ascending colon vessel, which suggests microbial metabolism provided for the release of these anthocyanins from the Amachi potato food matrix. Similarly, four anthocyanins were not noted in V1 for the Leona sample, which were pelargonidin 3-glucoside (*m*/*z* 433.11), petunidin 3-rhamnoside (*m*/*z* 463.12), cyanidin 3-(6-caffeoyl-glucoside) (*m*/*z* 611.14) and cyanidin 3-sophoroside (*m*/*z* 611.16). Interestingly, despite the overall several-fold higher total anthocyanin concentrations in the GI model vessels for the Amachi versus Leona cultivar, a seven-fold higher anthocyanin content in the Leona V5 was observed as compared to V5 anthocyanin concentrations for the Amachi cv. The above findings signify major differences in anthocyanin breakdown and release among the various digestive compartments between the two purple-fleshed potato cultivars. Such findings might be due to several cultivar matrix-mediated effects including: (a) enzymatic release of anthocyanins from the food matrix; (b) enzymatic and microbial-facilitated degradation of anthocyanins; (c) de-conjugation and subsequent re-conjugation of anthocyanins; (d) release and re-incorporation of anthocyanins within the fecal matrix; and (e) presence of other dietary constituents released during digestion on anthocyanin solubility.

Differentiating between different isobaric compounds could be done by looking at the retention time of the compound in some cases; for example, peonidin-3-caffeoyl-*p*-hydroxybenzoyl-sophoroside (*m*/*z* 907.22) was found to have a retention time of 13, while peonidin 3-*p*-hydroxybenzoyl-sophoroside 5-glucoside (*m*/*z* 907.25) was found at a retention time of 20. In other cases, isobaric compounds are not present always in the same vessels, potentially indicating that the method used for separation was accurate enough to isolate compounds that had very similar MW, such as cyanidin 3-sophoroside (MW: 611.16) and cyanidin 3-(6”-caffeoyl-glucoside) (MW: 611.14); the former is present in both the Amachi samples vessels V2 and V3, while the latter is only present in V3.

### 3.2. Antioxidant Activity

**Figure 2:**
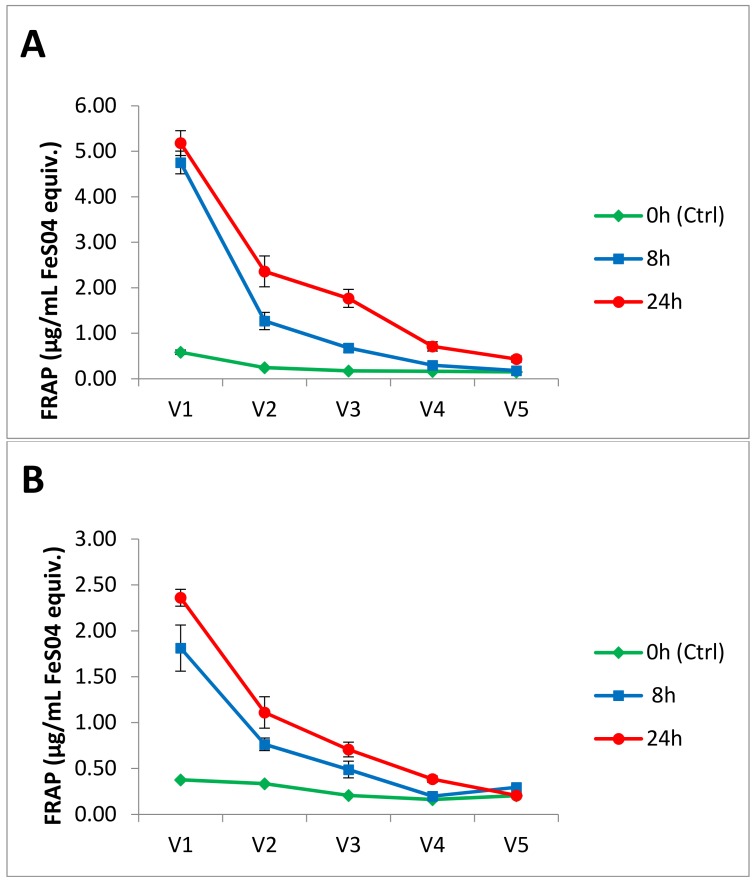
Time course of antioxidant capacity ferric reducing antioxidant power measures of digesta from gut model vessels following provision of a meal containing cvs. Amachi (**A**) and Leona (**B**) potato meals.

Samples were withdrawn from each of the five vessels of the GI model at *t* = 0 (before addition of potato meal) and *t* = 8 h and 24 h of digestion and antioxidant activity was assessed using the FRAP assay. As shown in Figure 2, antioxidant activity increased in vessels V1–V3 at 8 h, and further increased at 24 h of digestion. During exposure to intestinal V2 digestion, however, a decrease from initial values in the stomach V1 vessel was observed with both cultivars. This latter result coincides with previous studies showing a decrease in FRAP capacity of fresh apple [47] and anthocyanin-rich extracts and raw red cabbage shown after pepsin and pancreatin-bile digestion [8]. The antioxidant power of Amachi in all gut model vessels was approximately double that of Leona, which could be partly related to the several-fold higher content of both anthocyanins and chlorogenic acid (see Supplementary Materials, Figure S1) of Amachi in comparison to Leona. Degradation of the parent polyphenolic compounds including anthocyanins was likely responsible for the observed drop in antioxidant capacity seen in V2 vs. V1. A further decrease in antioxidant capacity was observed in V3, which was likely due to further diminishment of parent compounds resulting from gut microbial metabolism to generate secondary phenolic metabolites.

An increase in FRAP antioxidant activity in the V4 and V5 colonic reactors was only apparent at *t* = 24 h for the Amachi and this latter increase was only seen in V4 for Leona. Metabolic microbial breakdown of anthocyanins over a 24 h period appears to generate sufficient amounts of microbial metabolites to produce an improvement in antioxidant capacity. The latter result is supportive of the concept that microbial metabolites generated from ingested polyphenols can contribute to antioxidant capacity of human fecal fluid. In support of this contention, previous in vitro digestion studies have noted that a decrease in anthocyanin content is accompanied by an increase in antioxidant activity, which has been related to an increase in the concentrations of smaller molecular weight antioxidant phenolics [48]. The appearance of anthocyanin species in distal vessels of the GI model of both cultivars (Table 1) also supports this concept. Anthocyanins and their metabolites can, via antioxidant activity, provide protection of intestinal cells against oxidative stress in the gut, and hence alleviate gut inflammation [49], protect against colorectal cancer and generally enhance colorectal health [50]. It has been shown that the radical scavenging antioxidant ability of feces from healthy human subjects is 20-fold higher than that of plasma [51]. Desirable physiological effects following absorption may also be exerted by secondary metabolites generated from microbial polyphenol degradation. For instance, in a study where healthy volunteers consumed pomegranate juice for five days, microbial metabolites of polyphenols were detected in the plasma and urine, but the parent polyphenols were not [52].

### 3.3. Cytotoxicity and Cell Viability in Caco-2 and CCD-112CoN Cells

**Figure 3:**
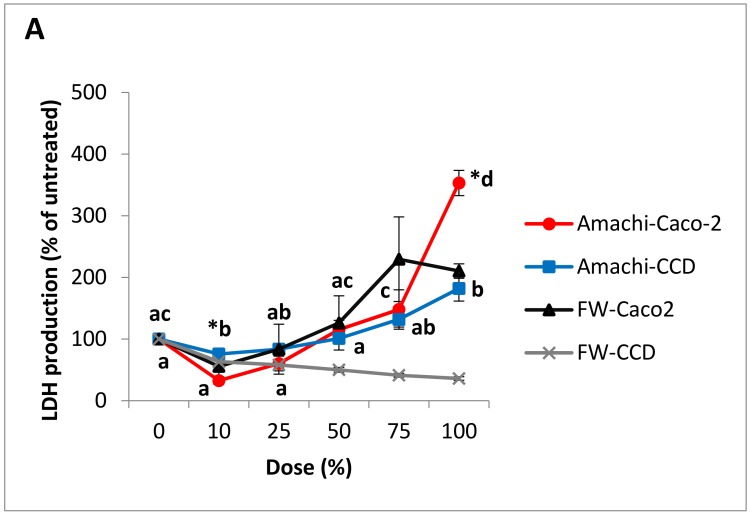

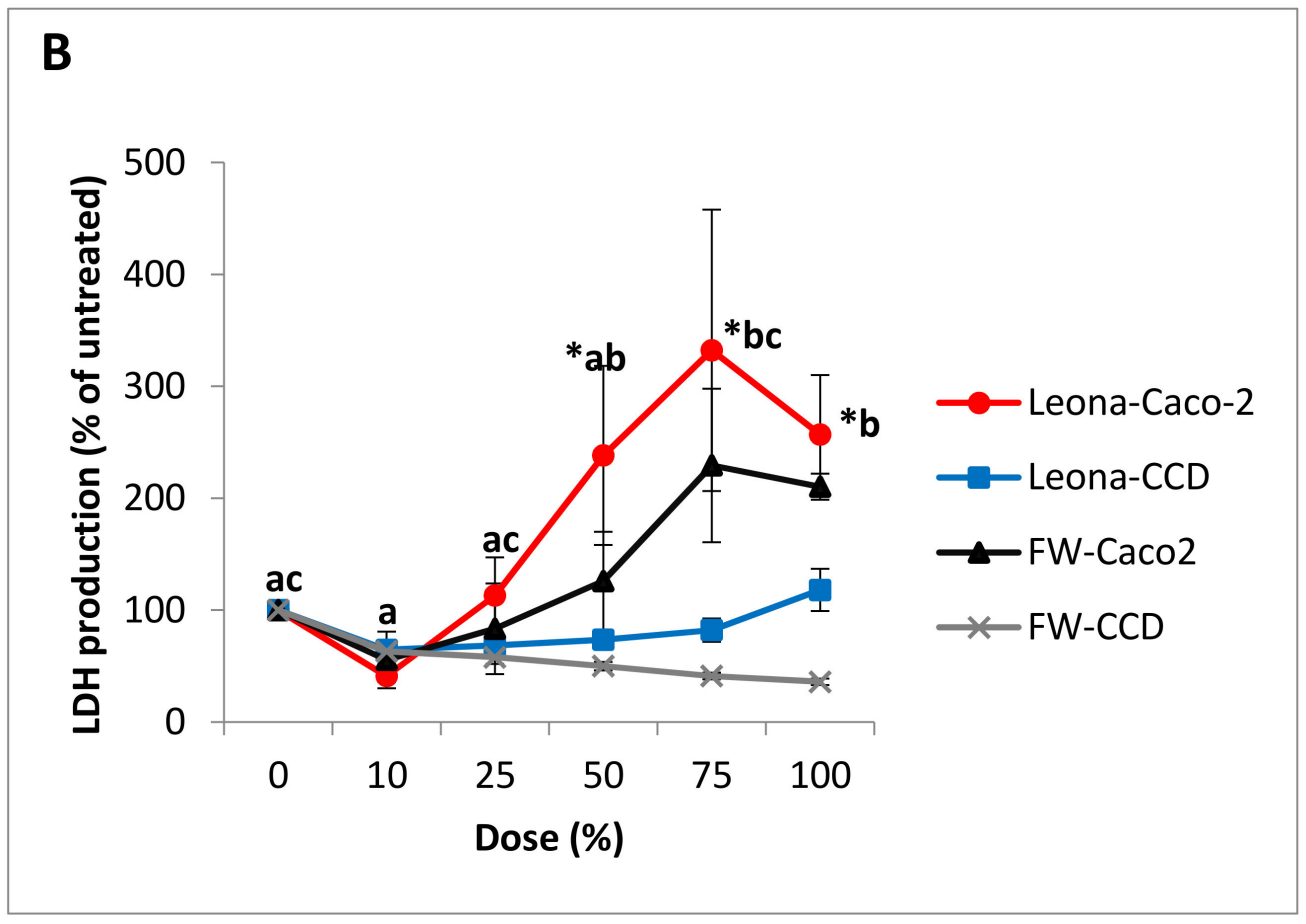
Effect of in vitro digests of cvs. Amachi (**A**) and Leona (**B**) on LDH production by Caco-2 and CCD-112CoN cells. Values are means ± SE of four independent experiments. Statistical analysis was performed via two-way ANOVA using cell line (Caco-2 versus CCD-112CoN) and dose (0%, 10%, 25%, 75% and 100%) as factors. Within each cell line, mean LDH values without a common letter are significantly different (*p* < 0.05). Between cell lines and within each dose, the symbol * represents a significant difference (*p* < 0.05) in the comparison between Caco-2 and CCD112-CoN cells at a specific digest dose.

Two human intestinal cell lines (the colorectal adenocarcinoma Caco-2 cell line and the normal colonic CCD-112CoN cell line) were used to evaluate the cytotoxic effects of FW obtained from colonic GI model digests of cvs. Amachi and Leona. Cytotoxicity was evaluated by the leakage of LDH in the culture media after 24 h treatment. LDH is a cytoplasmic enzyme retained by viable cells with intact plasma membranes, but it is released from necrotic cells with damaged membranes [53]. When cancer cells are exposed to high concentrations of compounds with anticancer properties, elevation of LDH in the cell culture medium is well established as a marker of necrosis [54]. The concentrations of anthocyanins and anthocyanin metabolites corresponding to doses of FW used are shown in Table 2. The LDH values were expressed as percent change relative to the untreated control that contained medium without digest. No changes in cytotoxicity were observed following treatment with FW alone in either cell line (Figure 3A,B).

**Figure 4:**
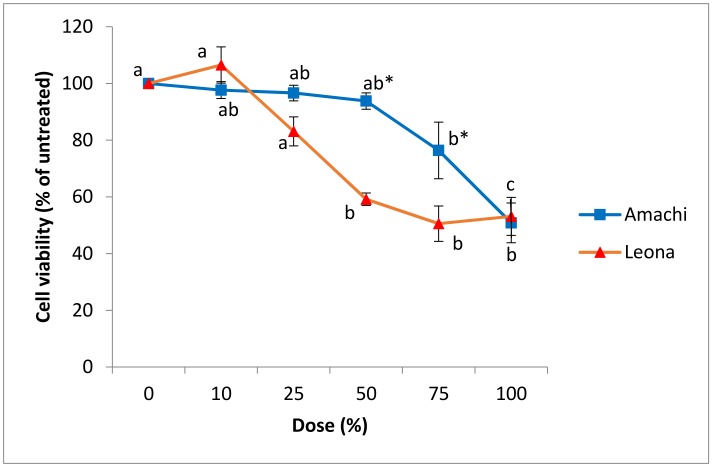
Effect of in vitro digests of cvs. Amachi and Leona on the viability of Caco-2 cells as assessed via the the 3-(4,5-dimethylthiazol-2-yl)-2,5-diphenyl tetrazolium bromide (MTT) assay. Values are means ± SE of four independent experiments. Means within cultivar treatment without a common letter are significantly different (*p* < 0.05). The symbol * represents the comparison between cvs. Amachi and Leona at a specific digest dose as significantly different (*p* < 0.05).

In Caco-2 cells, the colonic FW digest of Amachi at the 100% dose was associated with a significant (*p* < 0.05) increase in LDH production by 253% relative to untreated controls while the 10% dose showed significantly (*p* < 0.05) lower LDH release (Figure 3A). In comparison to Amachi digests, the digests of Leona exhibited greater cytotoxicity as the 75% dose in addition to the 100% dose was associated with a significant (*p* < 0.05) rise in LDH production of 232% and 157% compared to control cells, respectively (Figure 3B). The MTT viability assay findings were in concordance with the above results as FW from Amachi and Leona elicited a dose response decrease in cell viability in Caco-2 cells (Figure 4). In comparison to unexposed controls, Amachi FW showed significantly lower cell viability at the 75% and 100% dose while FW from Leona treatment showed lower cell viability at the 50%, 75% and 100% concentrations. Caco-2 cells demonstrated a significantly (*p* < 0.05) higher susceptibility to the Leona FW with an IC_50_ value of 1.02 mg/L versus 2.66 mg/L for FW from Amachi. The Leona FW exhibited significantly (*p* < 0.05) greater decrease in viability at the 50% and 75% doses than Amachi FW (Figure 4). The above findings agree with studies showing that simulated gastric and small intestine digestion of the high anthocyanin-containing Vitelotte cultivar was associated with decreased viability of Caco-2 cells in a concentration-dependent manner [27]. A dose-related inhibition of Caco-2 cell proliferation was also observed following exposure to freeze-dried extracts of anthocyanin-rich colored potatoes [25]. The present findings extend these latter results to demonstrate that colonic microbial metabolites of anthocyanin-rich colored potato meals decrease Caco-2 colonic cell viability in a cultivar-dependent manner. In that regard, despite the greater antioxidant capacity and higher anthocyanin content associated with the Amachi colonic digests, greater cytotoxicity was observed with the colonic FW from Leona. Such findings might be related to differences in anthocyanin profiles in the FW digests, particularly the approximately two-fold greater content of the aglycone cyanidin and petunidin anthocyanins in the Leona versus Amachi colonic reactors. Cyanidin, but not its glycosides, was shown to be a potent inhibitor of the growth of human colon carcinoma HT-29 cells [55] or HCT-116 [56] and Caco-2 [57] colon cancer cells. Aglycones such as cyanidin can readily be released following intestinal microbial beta-glucosidase-mediated hydrolysis of anthocyanins [58]. Thus, the presence of cyanidin as an intermediate microbial metabolite could provide chemopreventative properties towards colorectal cancer. Relatively greater concentrations of petunidin anthocyanins were related to pro-apoptotic effects of extracts of purple-fleshed potatoes in human HCT-116 colon carcinoma cells while antioxidant capacity and chlorogenic acid content was unconnected to the cancer cell inhibition [59]. Possible mechanisms of cytotoxicity could be related to anthocyanins acting as pro-oxidants leading to the generation of cytotoxic reactive oxygen species, which has been associated with anthocyanin exposure with tumor cells [18]. Oxidative stress has been indicated to lead to cancer cell death via induction of mitochondrial caspase-dependent and caspase-independent pathways of apoptosis, which have been shown following anthocyanin exposure [60]. It is also conceivable that unmeasured potato components and microbial metabolites were involved in the differing cytotoxicity and cell viability outcomes.

Cultivar differences were also noted in terms of cytotoxic effects of the FW digests on the non-tumorigenic CCD-112CoN cells. The Amachi digests were associated with enhanced cytotoxicity in the non-tumorigenic CCD-112CoN cells, which showed a significant (*p* < 0.05) increase in LDH release by 82% versus untreated cells at the 100% dose (Figure 3A). It has been well described that cytotoxic agents that promote necrosis in cancer cells can also induce toxicity to normal cells at higher concentrations [61]. On the other hand, no significant increase in LDH release was noted in the CCD-112CoN cells exposed to cv. Leona FW. The Caco-2 cancer cell line was generally more sensitive than the CCD-112CoN cells to cytotoxicity associated with FW from both cultivars. There was significantly (*p* < 0.05) higher LDH response to cv. Amachi digests in Caco-2 cells in comparison to CCD-112CoN cells at the 100% dose. The cv. Leona digests elicited significantly (*p* < 0.05) higher LDH release by Caco-2 versus CCD-112CoN cells at doses of 50% (238% versus 73.5% of untreated cells), 75%, (332% versus 82% of untreated cells) and 100% (257% versus 118% of untreated cells), respectively. Relative differences between tumor and normal colonic cell lines have been previously shown with anthocyanin-rich extracts, which demonstrated no significant toxicity towards normal colonic cells [62]. Such findings provide support for the concept that in vivo colonic exposure to digests of anthocyanin-rich potatoes can damage cancer cells with limited adverse effects on normal colonic cells. Further research is needed to measure the small molecular weight molecules in the colonic digests that could be tested on different tumorigenic and non-tumorigenic cell lines to better clarify the anticancer activities of the metabolites.

## 4. Conclusions

To our knowledge, this is the first study to report the effects of human simulated dynamic gastrointestinal digestion on anthocyanin profiles and antioxidant capacity in pigmented potatoes, as well as the effects of their FW digests on tumor and non-tumorigenic cell cytotoxicity and viability. Overall, the results of the present study revealed that the genetic background of the two purple potato cultivars analyzed led to major variances in composition and total content of anthocyanins throughout the GI model. An increase in antioxidant capacity with digestion time in all vessels was noted with both cultivars. This latter finding was likely due to antioxidant properties of the parent anthocyanin compounds in the upper intestinal vessels, whereas smaller phenolics could have contributed to the antioxidant effects in the colonic reactors. In our study, microbial metabolism can account for the anthocyanin degradation as seen by diminished concentrations of several anthocyanin species in the colonic vessels, which was particularly evident in the descending V5 colonic vessel of the Amachi cv. Our GI digestion model studies concurrently illustrate an increase in bioaccessibility associated with microbial biotransformation as shown by the initial appearance of several anthocyanin compounds throughout the colonic vessels in both tested potato cultivars. There was a greater than seven-fold higher concentrations of anthocyanins in the descending V5 colonic reactor of Leona versus Amachi, despite the several-fold higher total anthocyanin content in the overall GI model for the Amachi cv. The physiological relevance of this latter finding requires more investigation.

Interestingly, Leona FW showed relatively more potent effects on cytotoxicity and cell viability on colonic tumor cells. Unlike Amachi FW, the FW from Leona did not exhibit adverse effects on normal colonic cells. Further studies are needed to determine the identification of the bioactive components in the digesta and their mechanisms of action of cytotoxicity. The types of anthocyanins that contribute to the health promoting effects of purple potatoes require more research, including the bioavailability of anthocyanin metabolites. The examination of the digested anthocyanins and their metabolites generated from the gut model could be coupled with a cellular absorption model to assess for their bioavailabilities, which is presently in progress. This approach could help to identify the nature of bioactive anthocyanin compounds associated with the anti-cancer properties and other health attributes associated with the intake of purple potatoes. For example, antioxidant and antihypertensive effects of microwaved purple potatoes have been observed in hypertensive human subjects [63]. Insights arising from the above research can lead to objectives for potato breeding programs towards enhancing specific anthocyanins to develop varieties with chemopreventative properties.

## Figures and Tables

**Table 1:** Proposed identification of anthocyanin peaks in pigmented potato cvs. Amachi and Leona and their concentration in the samples exposed to human simulated intestinal digestion (mg/L) ^1^.

Measured Accurate Mass			Amachi					Leona				
(*m*/*z*) ^2^	Proposed Compound ^3^	RT	V1	V2	V3	V4	V5	V1	V2	V3	V4	V5
287.06	Cyanidin ^4^	3.1	0.38	-	-	-	-	0.40	-	-	0.31	0.48
301.03	Peonidin ^5^	4.8	0.47	-	0.35	-	-	-	-	-	-	-
433.11	Pelargonidin 3-glucoside	18	-	-	-	0.06	0.17	-	-	0.05	0.13	0.25
463.12	Petunidin 3-rhamnoside	18	-	-	-	-	-	-	-	0.03	0.10	0.16
583.14	Peonidin 3-*p*-hydroxybenzoyl-glucoside	5	0.16	-	-	-	-	0.07	0.14	0.14	0.22	-
610.16	Pelargonidin 3-feruloyl-glucoside	15	0.12	-	-	-	-	0.31	0.20	0.13	0.10	0.04
611.14	Cyanidin 3-(6-caffeoyl-glucoside)	15	-	-	0.17	-	-	-	0.09	0.02	0.03	0.04
611.16	Cyanidin 3-sophoroside	15	-	0.10	0.15	-	-	-	0.12	0.02	0.04	0.07
612.14	Peonidin 3-(6-*p*-coumaroyl-glucoside)	3.6	-	-	0.09	-	-	0.09	0.07	-	-	-
625.15	Peonidin 3-(6-caffeoyl-glucoside)	13	2.3	-	-	-	-	-	-	-	-	-
625.17	Petunidin 3-rutinoside	13	2.6	-	-	-	-	-	-	-	-	-
731.16	Cyanidin 3-(6-caffeoyl-6-*p*-hydroxybenzoyl-glucoside)	10	3.53	-	0.62	-	-	0.96	-	-	-	-
731.18	Cyanidin 3-*p*-hydroxybenzoyl-sophoroside	10	3.20	-	1.14	-	-	0.96	-	-	-	-
757.19	Cyanidin 3-*p*-coumaroyl-sophoroside	7	0.26	-	-	-	-	-	-	-	-	-
757.21	Pelargonidin 3-sophoroside-5-glucoside	7	0.28	-	-	-	-	0.07	-	-	-	-
771.21	Peonidin 3-(6”-*p*-coumaryl-sophoroside)	9	1.14	-	-	-	-	0.16	-	0.08	-	-
773.19	Cyanidin 3-(6”-caffeoyl-sophoroside)	15	-	-	0.53	-	-	-	-	-	-	-
773.21	Cyanidin 3-sophoroside-5-glucoside	7	-	-	-	1.11	-	-	-	-	-	-
787.20	Peonidin 3-(6″-caffeoyl-sophoroside)	7.7	4.55	-	-	0.23	-	0.26	-	-	-	-
787.22	Petunidin 3-rutinoside-5-glucoside	7.7	6.12	-	-	0.34	-	0.26	-	-	-	-
801.20	Peonidin 3-caffeoyl-feruloyl-glucoside	10	0.22	-	-	-	-	-	-	-	-	-
801.22	Peonidin 3-(6”-feruloyl-sophoroside)	10	0.30	0.07	-	-	-	-	-	-	-	-
893.21	Cyanidin 3-(6”-caffeoyl-6”-*p*-hydroxybenzoyl-sophoroside)	11	-	-	0.39	0.08	-	-	-	-	-	-
893.23	Cyanidin 3-*p*-hydroxybenzoyl-sophoroside-5-glucoside	11	-	-	0.39	0.07	-	-	-	-	-	-
903.23	Cyanidin 3-(6’,6”-dicoumaroyl-sophoroside)	20	2.20	-	-	-	-	-	-	-	-	-
907.22	Peonidin 3-caffeoyl-*p*-hydroxybenzoyl-sophoroside	13	-	-	0.04	0.02	-	-	-	-	-	-
907.25	Peonidin 3-*p*-hydroxybenzoyl-sophoroside-5-glucoside	20	-	-	0.02	-	-	-	-	-	-	-
917.27	Peonidin 3-*p*-coumaroyl-rutinoside-5-glucoside	22	16.42	-	-	-	-	0.06	-	-	-	-
919.25	Cyanidin 3-*p*-coumaroyl-sophoroside-5-glucoside	18	2.53	-	-	-	-	-	-	-	-	-
920.23	Cyanidin 3-caffeoyl-*p*-coumaroyl-sophoroside	18	1.11	-	-	-	-	-	-	-	-	-
931.25	Cyanidin 3-feruloyl-sophoroside-5-glucoside	20	0.09	-	-	-	-	-	-	-	-	-
933.26	Petunidin 3-*p*-coumaroyl-rutinoside-5-glucoside	20	132.79	0.40	0.07	0.01	0.02	0.15	0.13	-	0.04	0.26
949.23	Peonidin 3-dicaffeoyl-sophoroside	18	3.64	-	-	-	-	-	-	-	-	-
949.26	Peonidin 3-(6″′-caffeoyl-sophoroside)-5-glucoside	18	3.47	-	-	-	-	-	-	-	-	-
963.25	Peonidin 3-caffeoyl-feruloyl-sophoroside	20	4.39	-	-	-	-	-	-	-	-	-
963.27	Peonidin 3-(6”-feruloyl-sophoroside)-5-glucoside	20	4.45	-	-	-	-	-	-	-	-	-
	Total Anthocyanins Measured		196.72	0.57	3.96	1.92	0.19	3.75	0.75	0.47	0.97	1.3

^1^ Expressed as cyanidin 3-rutinoside equivalents after spiking samples with 10 μg/mL of keracyanin chloride. ^2^ Determined by liquid chromatography-electrospray ionization-time-of-flight analysis. ^3^ Identification based on previous literature data. ^4^ Cyanidin other RT 27 (Leona V4 and Leona V5). ^5^ Peonidin other RT 3.7 in Amachi V3.

**Table 2:** Concentrations of total anthocyanins corresponding to the doses of FW used in cell culture viability experiments, calculated from the concentrations in V3, V4 and V5 in Table 1.

Dose	Anthocyanin Concentration (mg/L Cyanidin 3-Rutinoside Equivalents)	
% FW in Cell Culture Media	Cv. Amachi	Cv. Leona
0%	0	0
10%	0.203	0.091
25%	0.507	0.228
50%	1.013	0.457
75%	1.520	0.685
100%	2.027	0.913

## Supplementary Materials

The following are available online at www.mdpi.com/2072-6643/9/9/953/s1, Figure S1: Time course of chlorogenic acid measurement of digesta from gut model vessels following provision of a meal containing different potato meals.
