# Supplementary material for: Effects of Simulated Human Gastrointestinal Digestion of Two Purple-Fleshed Potato Cultivars on Anthocyanin Composition and Cytotoxicity in Colonic Cancer and Non-Tumorigenic Cells
Source: Nutrients. 2017 Aug 29;9(9):953. doi: 10.3390/nu9090953 (PMC5622713; doi:10.3390/nu9090953)
Supplement: Supplementary file 1 [file nutrients-09-00953-s001.docx]

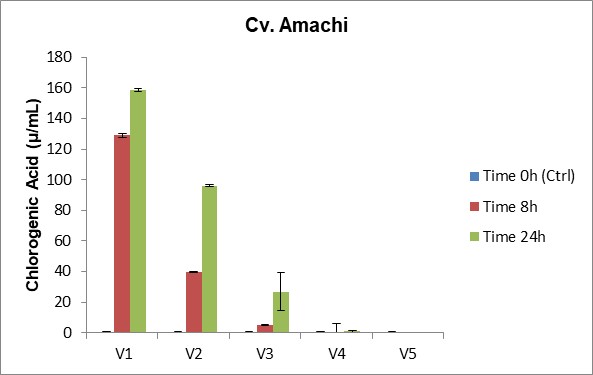


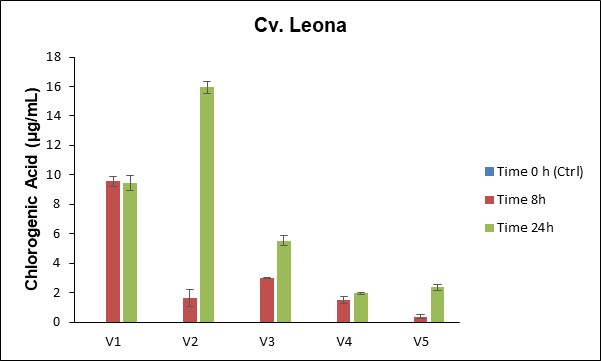


**Figure S1.** Time course of chlorogenic acid measurement of digesta from gut model vessels following provision of a meal containing different potato meals. V1 = stomach; V2 = small intestine; V3 = ascending colon; V4 = transverse colon; V5 = descending colon. Values are mean ± SD of two independent measurements.
